# Identification and Clinical Characteristics of Community-Acquired *Acinetobacter baumannii* in Patients Hospitalized for Moderate or Severe COVID-19 in Peru

**DOI:** 10.3390/antibiotics13030266

**Published:** 2024-03-16

**Authors:** Wilmer Silva-Caso, Giancarlo Pérez-Lazo, Miguel Angel Aguilar-Luis, Adriana Morales-Moreno, José Ballena-López, Fernando Soto-Febres, Johanna Martins-Luna, Luis J. Del Valle, Sungmin Kym, Deysi Aguilar-Luis, Dayana Denegri-Hinostroza, Juana del Valle-Mendoza

**Affiliations:** 1 School of Medicine, Research Center of the Faculty of Health Sciences, Universidad Peruana de Ciencias Aplicadas, Lima 15023, Peru; wilmer.silva@upc.pe (W.S.-C.); miguel.aguilar@upc.pe (M.A.A.-L.); johmarlu14@gmail.com (J.M.-L.); deysi3aguilar98@gmail.com (D.A.-L.); pcmedden@upc.edu.pe (D.D.-H.); 2 Division of Infectious Diseases, Guillermo Almenara Irigoyen National Hospital-EsSalud, Lima 15033, Peru; adri.mmm93@hotmail.com (A.M.-M.); jose.ballena@upch.pe (J.B.-L.); fernando.soto.f@upch.pe (F.S.-F.); 3 Facultad de Ciencias de la Salud, Universidad Tecnológica del Perú, Lima 15046, Peru; 4 Barcelona Research Center for Multiscale Science and Engineering, Departament d’Enginyeria Química, EEBE, Universitat Politècnica de Catalunya (UPC), 08034 Barcelona, Spain; luis.javier.del.valle@upc.edu; 5 Korea International Cooperation for Infectious Diseases, College of Medicine, Chungnam National University, Daejeon 35015, Republic of Korea; smkimkor@cnu.ac.kr

**Keywords:** community-acquired infections, *Acinetobacter baumannii*, OXA-51 gene

## Abstract

*Acinetobacter baumannii* has been described as a cause of serious community-acquired infections in tropical countries. Currently, its implications when simultaneously identified with other pathogens are not yet adequately understood. A descriptive study was conducted on hospitalized patients with a diagnosis of moderate/severe SARS-CoV-2-induced pneumonia confirmed via real-time RT-PCR. Patients aged > 18 years who were admitted to a specialized COVID-19 treatment center in Peru were selected for enrollment. *A. baumannii* was detected via the PCR amplification of the *bla*_OXA-51_ gene obtained from nasopharyngeal swabs within 48 h of hospitalization. A total of 295 patients with COVID-19 who met the study inclusion criteria were enrolled. *A. baumannii* was simultaneously identified in 40/295 (13.5%) of COVID-19-hospitalized patients. Demographic data and comorbidities were comparable in both *Acinetobacter*-positive and -negative subgroups. However, patients identified as being infected with *Acinetobacter* were more likely to have received outpatient antibiotics prior to hospitalization, had a higher requirement for high-flow nasal cannula and a higher subjective incidence of fatigue, and were more likely to develop *Acinetobacter*-induced pneumonia during hospitalization. Conclusions: The group in which SARS-CoV-2 and *A. baumannii* were simultaneously identified had a higher proportion of fatigue, a higher frequency of requiring a high-flow cannula, and a higher proportion of superinfection with the same microorganism during hospitalization.

## 1. Introduction

*A. baumannii* has gained importance as a pathogenic microorganism, predominantly in the hospital or healthcare setting [1]. In the hospital context, this microorganism is associated with multidrug-resistant infections, particularly ventilator-associated pneumonia. However, in tropical countries, characterized by hot and humid climates, *A. baumannii* has become a cause of serious community-acquired infections, with a higher prevalence during this month because of its natural resistance to desiccation [2,3,4]. 

Most cases of community-acquired *Acinetobacter baumannii* (CA-Ab) have been reported in Hong Kong, Singapore, Taiwan, South Korea, and North Australia [2]. However, there are reports in other regions, such as North America and recently Brazil [5]. The estimated incidence of CA-Ab-induced pneumonia with bacteremia in Australia and Taiwan is less than 10%, with no published data in other regions and a prevalence of 0.5% in culture-positive CAP patients [4,6].

Community-acquired pneumonia (CAP) has historically been attributed to *Streptococcus pneumoniae*, *Haemophilus influenzae*, *Mycoplasma pneumoniae*, *Staphylococcus aureus*, *Legionella* species, *Chlamydia pneumoniae*, and *Moraxella catarrhalis*. Empiric treatment of CAP has focused on these organisms and remains static despite potential differences regionally and changes in pathogen distribution over time. Furthermore, bacterial pneumonic infections can coexist with viral pneumonia as a simultaneous or secondary event [7,8].

During the COVID-19 pandemic, SARS-CoV-2 became the primary causative agent of community-acquired pneumonia [9,10]. This resulted in high morbidity and mortality among patients, in addition to the collapse of health systems owing to the demand for care due to a CAP outbreak on a global scale [9,10,11,12,13]. The simultaneous presence of *A. baumannii* as a community-acquired pathogen and SARS-CoV-2 in patients who develop moderate or severe pneumonia and its implications for clinical presentation and disease development remain unclear.

This study aimed to identify and describe the clinical characteristics of the simultaneous presence of community-acquired *A. baumannii* and SARS-CoV-2 in patients diagnosed with COVID-19 who were treated for moderate or severe pneumonia.

## 2. Results

This study included 295 patients with a confirmed diagnosis of COVID-19 who were admitted for moderate or severe pneumonia during the first wave of the pandemic. In 40 (13.5%) analyzed samples, *A. baumannii* was identified in the presence of SARS-CoV-2. The group of patients in whom SARS-CoV-2 and *A. baumannii* were identified at the same time had a similar distribution in terms of sex and age to the group that presented only SARS-CoV-2 infection.

In our analysis, no significant differences were found between comorbidities (high blood pressure, diabetes mellitus, obesity, asthma, and others described), the CURB-65 index, and the majority of the symptoms and signs evaluated, except for the fact that those patients in whom SARS-CoV-2 and *A. baumannii* were simultaneously identified had a higher proportion of fatigue at clinical presentation (65% vs. 47.8%; *p* = 0.044). In both groups, the values for the median number of days from symptom onset to hospitalization were similar (7 days) (Table 1).

Likewise, no statistically significant differences were found between the laboratory parameters taken upon a patient’s admission to a hospital. However, the median leukocyte count was higher in patients in whom SARS-CoV-2 and *A. baumannii* were identified than in those in whom only SARS-CoV-2 was identified (10.5 vs. 8.9 × 10^9^ mL, *p* = 0.055). In relation to the treatments received, previous use of antimicrobials on an outpatient basis was more frequent among patients in whom both pathogens were identified (82.5% vs. 67.5%, *p* = 0.055). In individuals in whom both pathogens were identified simultaneously, a greater requirement for a high-flow cannula was evident during hospitalization (15% vs. 5.5%, *p* = 0.038) (Table 2).

Patients in whom SARS-CoV-2 and *A. baumannii* were identified simultaneously upon hospital admission had a higher frequency of superinfection by the same microorganism during their hospitalization (10% vs. 1.6%, *p* = 0.014) (Table 3). No statistically significant differences were found in other clinical results. Hospital outcomes such as mortality, sepsis, acute respiratory distress syndrome, and heart failure, among others, showed no differences between the groups. It is worth mentioning that although the presence of respiratory acidosis and the mean number of days of hospitalization did not result in a significant difference, the frequency was slightly higher in the group in which both pathogens were identified simultaneously.

## 3. Discussion

The etiological profile of CAP changed during the COVID-19 pandemic. Prior to this, some estimates indicated that close to 70% of CAP cases could be of bacterial etiology. However, in routine clinical practice, approximately 60% of pneumonia cases do not have an identified microbiological diagnosis [14,15]. Among the factors that explain this statement, we can point out the extraordinary etiological heterogeneity and existence of diagnostic limitations in health facilities, especially in undeveloped countries [7,16,17]. In this context, the use of molecular diagnostic platforms has enabled the identification of *A. baumannii* as a community pathogen causing CAP, which has been reported with increasing frequency and high mortality since before the COVID-19 pandemic. Diseases caused by this microorganism are fostered by the environmental persistence of this bacterium, resistance to desiccation, and evasion of the immune system [18,19].

Regarding the identification of *A. baumannii* as a community-acquired pathogen in patients with COVID-19, we found 40 (13.5%) positive cases in the samples analyzed. This represents more than double the frequency reported in other studies that simultaneously identified community bacteria in patients with COVID-19. In these studies, pathogen analysis was performed using urine, blood, and respiratory tract samples [20]. Although our study found that 85% of pneumonia cases had SARS-CoV-2 positivity and *A. baumannii* negativity, it is known that the prevalence of bacterial pneumonia acquired in a community where only *A. baumannii* is identified as an emerging bacterial pathogen ranges from 0% to 6%. However, there are studies where a higher frequency of isolation of other bacteria such as *S. aureus* has been reported simultaneously with COVID-19 during hospital admission during the pandemic [20,21,22].

The clinical presentation of patients with a mono-infection of SARS-CoV-2 has been extensively documented, particularly compared to those with simultaneous bacterial infections. Common symptoms upon hospital admission included cough, dyspnea, and fever, occurring at similar frequencies in both the mono-infection and co-infection groups [22]. However, expectoration seems uncommon in patients in whom pathogens such as *Mycoplasma pneumoniae* and/or *Chlamydia pneumoniae* were simultaneously identified [23]. In cases of co-infection with *A. baumannii* and SARS-CoV-2, our study highlights increased fatigue and a higher requirement for high-flow oxygen therapy at admission than for mono-infected individuals. Interestingly, none of the co-infected patients exhibited anosmia. These general clinical characteristics contrast with reports of CAP induced by *A. baumannii* that report continuous fever, intermittent cough with bloody sputum, dyspnea, chest pain, and the rapid deterioration of the patient [24,25,26].

The present study revealed that 70% of all hospitalized patients had received prior outpatient antibiotics, some of which were broad-spectrum agents that could predispose them to higher rates of community-acquired resistance and promote the acquisition of historically considered nosocomial pathogens. Additionally, the group of patients in whom *A. baumannii* and SARS-CoV-2 were identified within the first 48 h of hospital admission showed a higher proportion of superinfection caused by the same species of microorganisms during hospitalization. Although the initial pathogenic role of this bacterium during hospital admission cannot be precisely determined, it is known that even colonization by multidrug-resistant (MDR) strains is associated with higher mortality, longer hospital stays, and increased costs in intensive care units [27]. Since the onset of the COVID-19 pandemic and throughout its course, the irrational use of antimicrobial drugs by the general population has been described and warned against. In this regard, prehospitalization consumption of antimicrobials from the carbapenem family, including imipenem and meropenem, as reported in our study, is described as a risk factor for the acquisition of *A. baumannii* [28]. Among patients infected with *Acinetobacter*, the rate of outpatient antibiotic use was significantly higher, supporting this possibility.

We did not find differences in mortality between the group of patients with mono-infections and those co-infected with *A. baumannii*; however, a trend of longer hospital stay was highlighted in this group, particularly in patients requiring intensive care. Timely identification of these pathogens may be crucial in reducing CAP-Ab mortality from 40–64% to 11% when molecular support tests such as next-generation sequencing are used in combination with initial clinical decision making [25]. Given these findings, the implementation of a treatment protocol including antibiotics with a spectrum covering *Acinetobacter* species should be evaluated and adapted, especially in other tropical areas outside Northern Australia, where guidelines recommend the administration of 1 g of meropenem three times a day intravenously plus the intravenous administration of 500 mg of azithromycin daily for the empirical treatment of severe pneumonia [2]. Additionally, rapid detection of carbapenem-resistant *A. baumannii* (CRAB) carriers is recommended, as it allows for earlier implementation of preventive measures for reducing transmission in outbreak settings [29]. Viral infections such as SARS-CoV-2 may lead to dysbiosis of the nasopharyngeal microbiota and increase the risk of co-infections [30].

The presence of multidrug-resistant bacteria, such as *A. baumannii*, alongside a highly contagious virus, such as SARS-CoV-2, poses additional challenges for infection control in hospital settings and requires coordinated treatment decisions among medical teams. Moreover, conducting local epidemiological investigations is warranted to understand the transmission dynamics of these pathogens in the community and their impact on hospital epidemiology, thus influencing infection prevention and control strategies [31,32].

Our findings are local in scope, and with the existing data and our limitations, it is not possible to predict whether changes in bacterial prevalence will occur for this particular infection. However, there are studies that seek to predict the risk of bacterial infections and their impact on prevalence in critical care settings using patient data found in a patient’s medical history through random prediction models [33]. These prediction models have not been applied to infections caused by CA-*Acinetobacter*. Despite this, retrospective studies carried out in tropical countries recommend continuous specific surveillance of CA-*Acinetobacter* by health authorities [34].

Our study has several limitations. First, a specific site with a higher sensitivity for obtaining surveillance samples of *A. baumannii* has not been established. Although nasopharyngeal sampling during the early hospitalization period has been considered [35,36], it was not possible to determine whether the *A. baumannii* isolates belonged to the same cluster. Additionally, environmental screening studies were not conducted, nor was the impact of hospital disinfection programs in the isolation rooms considered. Moreover, this was a single-center study, and the prevalence rates of *Acinetobacter* vary regionally, while pre-pandemic incidence rates are unavailable, making it unclear if the current findings represent longitudinal trends or transient effects of COVID-19. Methodologically, our study has some bias. Selection bias was present owing to the absence of an ideal control group, complicating comparisons of simultaneous identification incidence in COVID-19 patients and the general population. Confounding bias may have influenced the results due to unconsidered factors, such as age, sex, or pre-existing conditions. Information bias arose from the difficulty of distinguishing the presence of *A. baumannii* as coinfection from incidental colonization. Despite these limitations, potentially impacting result interpretation and conclusion validity, our findings highlight complications, identify preliminary associations, and guide future research.

## 4. Materials and Methods

### 4.1. Study Design

A descriptive study was conducted on hospitalized patients diagnosed with moderate/severe SARS-CoV-2 pneumonia confirmed via real-time RT-PCR. Individuals over 18 years of age who entered a high-complexity hospital equipped to care for COVID-19 patients during the first wave of the pandemic between July and November 2020 were selected. Participants were consecutively enrolled until 295 individuals met the inclusion criteria.

Patients who were enrolled upon hospital admission had no history of hospitalization or had been in long-term care or nursing homes within 90 days prior to admission. This information was verified according to each patient’s anamnesis and clinical history. The patients were recruited during the first wave of the COVID-19 pandemic and had been under home isolation imposed by health authorities.

### 4.2. Informed Consent Was Obtained at Hospital Admission

Minors under 18 years of age, pregnant women, individuals who refused to participate, and individuals who, due to their entry status, could not sign the informed consent form were not considered for this study.

### 4.3. Definitions

Moderate SARS-CoV-2 pneumonia was defined as the presence of clinical signs of pneumonia (fever, cough, dyspnea, and respiratory distress) but without signs of severe pneumonia (O2 saturation ≥ 90%). Severe pneumonia was defined as the presence of the aforementioned clinical signs plus one of the following: respiratory rate > 30/min, severe respiratory distress, or O2 saturation < 90% [37]. ARDS is defined as acute diffuse inflammatory lung injury, leading to increased pulmonary vascular permeability, increased lung weight, and loss of aerated lung tissue [38].

To define coinfection and superinfection, the standards of the Centers for Disease Control and Prevention (CDC, Atlanta, GA, USA) were used, where coinfection is defined as an infection that occurs concurrently with the initial infection, while superinfections are those that follow a previous infection, especially when they are caused by resistant microorganisms or have become resistant to previously used antibiotics [39,40]. In the present study, superinfection by *A. baumannii* was established based on its definition and what is indicated as a healthcare-associated infection (HAI), for which the clinical presentation, images supporting the diagnosis, and laboratory criteria were assessed. National Health Safety Network (NHSN) criteria were followed, and data on ventilator-associated pneumonia (VAP), catheter-related bacteremia, and catheter-associated urinary tract infection were included [41].

### 4.4. Obtaining the Samples and Extraction of Nucleic Acids

Samples were obtained within the first 48 h after admission to the hospital. Nasopharyngeal swab tests were performed according to the CDC methodology on individuals who were in the hospitalization wards and intensive care units (ICU) designated for the care of patients with COVID-19.

Genetic material was extracted using 140 µL of samples. The QIAamp Nucleic Acid Isolation Kit (QIAGEN^®^, Düsseldorf, Germany) was used according to the manufacturer’s instructions. Eighty microliters of eluted DNA was obtained after extraction, and the amplification process was continued to determine the presence of *A. baumannii*.

PCR amplification

Real-time polymerase chain reaction (real-time PCR)

To detect *A. baumannii* [42,43], the following primers were used, as described by Chuang et al.:

OXA-51-F TTTAGCTCGTCGTATTGGACTTGA

OXA-51-R CGGAGAAGGACCCACCAGCCAAAA

OXA-51-Sonda FAM-TGGCAATGTAGATATCGGTACCCAAGTC-TAMRA

A LightCycler FastStart DNA Master HybProbe (Roche Applied Science, Penzberg, Germany) was used for the assay. The reaction mixture was 7.6 µL of nuclease-free water, 2.4 µL of buffer solution with 25 mM of Mg^2+^, 2.0 µL of Faststart Enzyme, 1 µL of each OXA-51-specific primer in 1 mM concentration, 1 µL of probe, and 5 µL of DNA. The amplification conditions were 95 °C for 10 min, followed by 55 cycles at 95 °C for 5 s, 60 °C for 5 s, and 72 °C for 15 s. LightCycler 2.0 was used, and data were analyzed with LightCycler 4.1 software (Roche Diagnostics, Mannheim, Germany) [44].

### 4.5. Data Analysis

Data and variables were collected by reviewing the hospital electronic medical records. The information obtained was compiled in a database stored in Microsoft Excel v.2016 using the double-typing technique.

### 4.6. Statistical Analysis

Statistical comparisons were made between patients for whom a simultaneous molecular identification of *A. baumannii* and SARS-CoV-2 was made versus the group for whom only SARS-CoV-2 was isolated. The chi2 test or Fisher’s exact test was used for dichotomous variables, and the *t*-test or Mann–Whitney test was used for continuous variables. Statistical analysis was performed using Stata SE 15.0 software for Windows College Station, TX, USA. Statistical significance was set at *p* value ≤ 0.05.

## 5. Conclusions

Patients in whom *A. baumannii* and SARS-CoV-2 were simultaneously detected had a higher proportion of fatigue at clinical presentation. Previous use of antimicrobials on an outpatient basis was more frequent in patients in whom both pathogens were identified, and a greater requirement for a high-flow cannula was evident. There was a higher frequency of superinfection with the same microorganism during hospitalization.

## Figures and Tables

**Table 1:** Demographic characteristics and symptoms upon admission.

	Total (*n* = 295)	COVID-19/*Acinetobacter baumannii* (−) (*n* = 255)	COVID-19/*Acinetobacter baumannii* (+) (*n* = 40)	*p* Value
Gender				
Male	209 (70.8)	181 (71.0)	28 (70.0)	0.899
Female	86 (29.2)	74 (29.0)	12 (30.0)	
Age				
Media/SD	58.0 ± 14.0	58.0 ± 13.9	57.9 ± 14.5	0.967
Comorbidities				
Hypertension	79 (26.8)	68 (26.7)	11(27.5)	0.912
Diabetes	66 (22.4)	61 (23.9)	5 (12.5)	0.107
Obesity	55 (18.6)	47 (18.4)	8 (20.0)	0.813
Asthma	12 (4.1)	12 (4.7)	0 (0.0)	0.381
Chronic coronary heart disease	12 (4.1)	10 (3.9)	2 (5.0)	0.670
Cancer	7 (2.4)	6 (2.4)	1 (2.5)	1.000
CKD *	4 (1.4)	4 (1.6)	0 (0.0)	1.000
Others	56 (18.9)	50 (19.6)	6 (15.0)	0.490
Symptoms				
Cough	215 (72.9)	184 (72.2)	31 (77.5)	0.480
Dyspnea	220 (74.6)	193 (75.7)	27 (67.5)	0.269
Fever	180 (61.0)	156 (61.2)	24 (60.0)	0.887
Fatigue	148 (50.2)	122 (47.8)	26 (65.0)	0.044
Odynophagia	39 (13.2)	32 (12.5)	7 (17.5)	0.390
Headache	35 (11.9)	30 (11.8)	5 (12.5)	0.894
Nausea/vomiting	18 (6.1)	16 (6.3)	2 (5.0)	1.000
Diarrhea	20 (6.8)	16 (6.3)	4 (10.0)	0.328
Expectoration	27 (9.2)	24 (9.4)	3 (7.5)	1.000
Anosmia	11 (3.7)	11 (4.3)	0 (0.0)	0.371
Days since symptom onset *	7 (5–10)	7 (5–10)	7 (5–8.5)	0.613
CURB 65 *	1 (0–2)	1 (0–2)	1 (0–2)	0.162

* Median (Interquartile Range). SD, Standard deviation; CKD, Chronic Kidney Disease; CURB 65, Community-Acquired Pneumonia Severity Score.

**Table 2:** Laboratory and radiological parameters and medications upon patient admission.

	Total (*n* = 295)	COVID-19/*Acinetobacter baumannii* (−) (*n* = 255)	COVID-19/*Acinetobacter baumannii* (+) (*n* = 40)	*p* Value
Laboratory parameters *				
Hemoglobin (g/dL)	14.2 (13.1–15.4)	14.2 (13.0–15.4)	14.7 (13.1–15.3)	0.717
Leukocytes (×10^9^ mL)	9.1 (7.9–12.3)	8.9 (6.8–11.8)	10.5 (7.7–14.4)	0.055
Lymphocytes (absolute count)	820 (504–1290)	806 (502.0–1247.0)	926.5 (526–1633.5)	0.312
Platelets (×10^9^ mL)	270 (202–350)	270 (202–349)	262.5 (181–353.5)	0.547
ALT (U/L)	49 (26.5–88)	47 (26–88)	50.5 (29. 5–88.5)	0.702
Creatinine (mg/dL)	0.7 (0.6–0.9)	0.7 (0.6–0.9)	0.7 (0.6–0.9)	0.791
C-reactive protein (mg/L)	90 (56–210)	107 (57.6–219)	71.65 (34.3–154)	0.072
LDH (U/L)	298 (242.5–378.5)	298.0 (244–368)	307 (249.5–382)	0.652
Procalcitonin (ng/mL)	0.1 (0.1–0.3)	0.1 (0.1–0.2)	0.1 (0.1–0.3)	0.721
D-Dimer (µg/mL)	0.6 (0.4–1.2)	0.6 (0.4–1.2)	0.8 (0.7–0.9)	0.555
Troponin (ng/mL)	0.006 (0.001–0.10)	0.006 (0.001–0.01)	0.085 (0.006–0.012)	0.267
Ferritin (ng/mL)	664.5 (346–1220)	659.5 (359.5–1219)	669 (315–1249)	0.970
CPK (U/L)	55 (33–88)	42 (31–92)	42 (33–62)	0.694
PT (s)	10.9 (10.4–11.5)	10.9 (10.4–11.5)	10.9 (10.5–11.4)	0.726
Score-radiological				
Media/SD	5.92 ± 1.55	5.92 ± 1.81	5.9 ± 2.2	0.946
Treatment				
Antibiotics prior to admission	205 (69.5)	172 (67.5)	33 (82.5)	0.055
Azithromicin	95 (46.3)	84 (48.8)	11 (33.3)	0.128
Ceftriaxone	141 (68.8)	117 (68.0)	24 (72.7)	0.685
Imipenem	36 (17.6)	29 (16.8)	7 (21.2)	0.617
Meropenem	20 (9.8)	15 (8.7)	5 (15.2)	0.331
Piperacilin/tazobactam	29 (14.1)	21 (12.2)	8 (24.2)	0.098
Vancomycin	28 (13.7)	22 (12.8)	6 (18.2)	0.411
Doxycycline	6 (2.9)	4 (2.3)	2 (6.0)	0.248
Ciprofloxacin	5 (2.4)	4 (2.3)	1 (3.0)	0.588
Levofloxacin	1 (0.5)	1 (0.6)	0 (0.0)	1.000
Amoxicillin	1 (0.5)	1 (0.6)	0 (0.0)	1.000
Amikacin	1(0.5)	1 (0.6)	0 (0.0)	1.000
Linezolid	1 (0.5)	1 (0.6)	0 (0.0)	1.000
Trimethoprim/sulfamethoxazole	1 (0.5)	0 (0.0)	1 (3.0)	0.161
Clindamycin	2 (0.9)	2 (1.16)	0 (0.0)	1.000
Cefepime	2 (0.9)	2 (1.16)	0 (0.0)	1.000
Ceftazidime	1 (0.5)	1 (0.6)	0 (0.0)	1.000
Tigecycline	3 (1.5)	3 (1.7)	0 (0.0)	1.000
Colistin	3 (1.5)	3 (1.7)	0 (0.0)	1.000
Dexamethasone	250 (84.7)	216 (84.7)	34 (85.0)	0.962
Hydroxychloroquine	3 (1.0)	3 (1.2)	0 (0.0)	1.000
Ivermectin	24 (8.1)	20 (7.8)	4 (10.0)	0.548
Binasal cannula	161 (54.6)	143 (56.1)	18 (45.0)	0.191
Reservoir mask	111 (37.6)	95 (37.3)	16 (40.0)	0.739
High flow nasal cannula	20 (6.8)	14 (5.5)	6 (15.0)	0.038
Mechanic ventilation	20 (6.8)	17 (6.7)	3 (7.50)	0.845
Norepinephrine	21 (7.1)	17 (6.7)	4 (10.0)	0.504
Hemodialysis	3 (1.0)	3 (1.2)	0 (0.0)	1.000

* Median (Interquartile Range) ALT = Alanine aminotransferase; LDH = Lactate dehydrogenase; CPK = Creatine phosphokinase; PT = prothrombin time.

**Table 3:** Clinical outcomes of the patients.

Hospital Outcomes	Total (*n* = 295)	COVID-19/*Acinetobacter baumannii* (−) (*n* = 255)	COVID-19/*Acinetobacter baumannii* (+) (*n* = 40)	*p* Value
Sepsis	80 (27.1)	65 (25.5)	15 (37.5)	0.112
ARDS *	60 (20.3)	48 (18.8)	12 (30.0)	0.103
Heart failure	25 (8.5)	22 (8.6)	3 (7.5)	1.000
Septic shock	24 (8.1)	18 (7.1)	6 (15.0)	0.113
Coagulopathy	17 (5.8)	15 (5.9)	2 (5.0)	1.000
Acute myocardial injury	12 (4.1)	12 (4.7)	0 (0.00)	0.381
Acute kidney injury	30 (10.2)	26 (10.2)	4 (10.0)	1.000
Respiratory acidosis	28 (9.5)	21 (8.2)	7 (17.5)	0.079
Admission to ICU U	29 (9.8)	24 (9.4)	5 (12.5)	0.567
Days in ICU (median/IQR)	11 (6–21)	9.5 (4–19)	17 (9–29)	0.153
Days on mechanical ventilation (median/IQR)	11 (1–19)	11 (1–19)	17 (1–45)	0.641
Days of hospitalization (median/IQR)	10 (7–15)	10 (7–15)	12 (7.8–22.5)	0.080
Death	59 (20.0)	49 (19.2)	10 (25)	0.395
Superinfection with *Acinetobacter baumannii* during hospitalization	8 (2.7)	4 (1.6)	4 (10)	0.014

* ARDS, acute respiratory distress syndrome; ICU = intensive care unit. IQR = interquartile range.

## Data Availability

The data supporting the reported results are available from the corresponding author upon reasonable request.
